# Intracerebral Hematoma in Patients With Impella Ventricular Assist Device Placement for Cardiogenic Shock: Report of Three Cases

**DOI:** 10.7759/cureus.48863

**Published:** 2023-11-15

**Authors:** Mitsuki Horio, Daina Kashiwazaki, Takahiro Tomita, Kunitaka Maruyama, Saori Hamada, Emiko Hori, Makiko Nakamura, Koichiro Kinugawa, Satoshi Kuroda

**Affiliations:** 1 Department of Neurosurgery, Toyama University Hospital, Toyama, JPN; 2 Department of Internal Medicine, Toyama University Hospital, Toyama, JPN

**Keywords:** morbidity, mechanical circulatory support, intracerebral hemorrhage, impella, hematoma evacuation, bleeding

## Abstract

Despite the clear benefits of Impella in patients with cardiogenic shock, bleeding is a possible complication. Herein, we report three cases of intracerebral hemorrhage in patients with Impella implantation for cardiogenic shock, which were treated with hematoma evacuation. We present the clinical features, diagnosis, and management (hematoma evacuation) of patients with the Impella device (Abiomed, Danvers, Massachusetts) who developed intracerebral hemorrhage. Case one was a 56-year-old man who presented with chest pain and loss of consciousness, was diagnosed with acute myocardial infarction, and underwent urgent percutaneous coronary intervention and Impella placement. After eight days, the patient developed anisocoria. Computed tomography revealed a left intracerebral hemorrhage. An emergency hematoma evacuation was successfully performed (intraoperative blood loss: 2600 mL). Case two was a 54-year-old male who presented with persistent chest pain and loss of consciousness, was diagnosed with acute myocardial infarction, and underwent an emergency percutaneous coronary intervention with Impella implantation and venoarterial extracorporeal membrane oxygenation. The patient developed intracerebral hemorrhage after 26 days. Hematoma evacuation was successfully performed (intraoperative blood loss: 380 mL). Case three was a 52-year-old male who presented with dyspnea and hypotension, was diagnosed with dilated cardiomyopathy, and underwent Impella implantation and venoarterial extracorporeal membrane oxygenation, followed by which the patient developed subcortical hematoma. An emergency hematoma evacuation was performed (intraoperative blood loss: 3205 mL). The patient died 14 days after admission. Intracerebral hemorrhage is a potential cause of morbidity associated with Impella placement. Although hematoma evacuation is optimal, the bleeding tends to increase.

## Introduction

Historically, cardiogenic shock has been treated with vasoactive medications and intra-aortic balloon pumps (IABPs) [1]. The Impella device (Abiomed, Danvers, Massachusetts) is a transaortic valvular pump that directly displaces blood from the left ventricle into the aorta, decreases the workload and myocardial oxygenation demand while increasing the mean arterial pressure, and provides direct circulatory support. Of the temporary mechanical circulatory support (MCS) devices, the Impella is increasingly used as an alternative to the IABP for hemodynamic support in patients with cardiogenic shock because of its superior ability to augment cardiac output and reduce shock severity parameters; however, it has yet to show a significant effect on reducing 30-day mortality. [2] Despite its clear benefits [3,4], bleeding is a possible complication because of anticoagulant usage, antiplatelet agents intake, and coagulopathy [4-6], and little is known about surgical hematoma evacuation in patients with Impella support.

Herein, we report three consecutive cases of intracerebral hemorrhage (ICH) developed during Impella support, which were managed with surgical hematoma evacuation. The aim of this report is to share important notice when hematoma evacuation for the patients under Impella support.

## Case presentation

Case one

**Figure 1:**
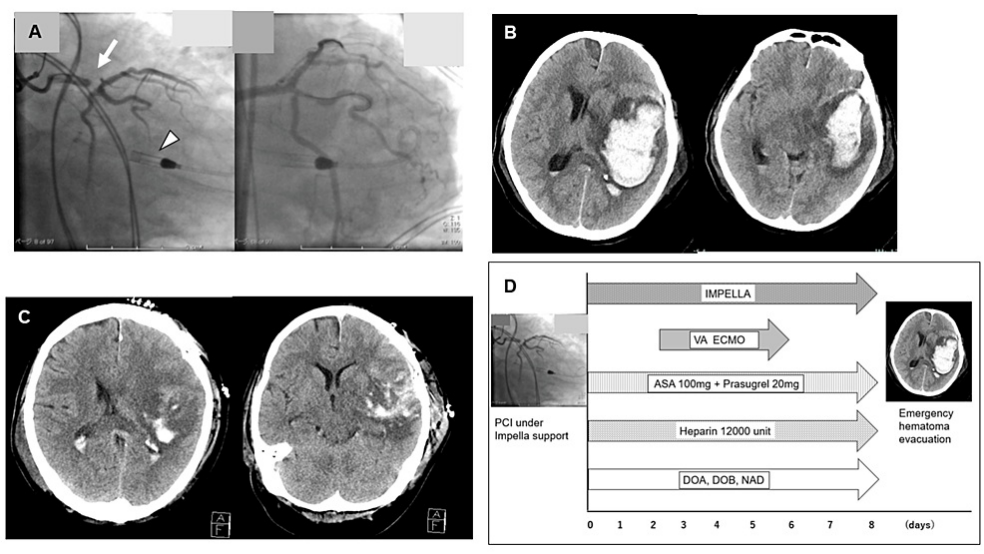
Clinical course of case one A) Images of the left anterior descending artery before (left) and after (right) percutaneous coronary intervention: Severe stenosis of the left anterior descending artery was treated by percutaneous transluminal angioplasty and stent placement. The Impella was implanted to treat cardiogenic shock; B) Intracerebral hemorrhage in the left frontal and temporal lobes. The hematoma volume is 59.3 mL; C) Computed tomography after emergency hematoma evacuation shows that the hematoma had been removed; D) Graph showing the treatment process for case one from admission to the onset of intracerebral hemorrhage VA ECMO - venoarterial extracorporeal membrane oxygenation; PCI - percutaneous coronary intervention; ASA - acetylsalicylic acid

A 56-year-old male presented with sudden chest pain and loss of consciousness as symptoms of acute myocardial infarction (AMI) and was in cardiogenic shock. Despite administering inotropes and vasopressors, systolic blood pressure and mixed venous oxygen saturation were low, which led to the use of axillary Impella 5.0. Electrocardiography (ECG) showed extensive myocardial ischemia. Urgent percutaneous coronary intervention (PCI) was performed for the 99% stenosis of the left anterior descending artery (Figure 1A)

**Figure 2:**
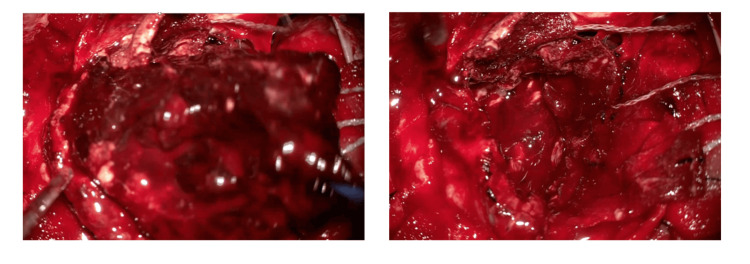
Intraoperative photo of case one This photo shows hematoma evacuation (left panel). A large hematoma was removed. This photo showed porencephaly after hematoma removal (right panel).

Dual antiplatelet therapy (acetylsalicylic acid (ASA) 100 mg and prasugrel 20 mg) and intravenous heparin injection were started, followed by Impella placement. Eight days after the PCI, right hemiparesis and anisocoria were observed. Cranial computed tomography (CT) revealed left subcortical hemorrhage with midline shift (hematoma volume: 59.3 mL) (Figure 1B). Protamine has been used as the principal neutralizing agent of heparin. An experienced neurosurgeon performed an emergency craniotomy for hematoma evacuation. There was primary hemorrhage, and hemostasis was difficult to establish (intraoperative blood loss: 2600 mL). Intraoperative transfusion with red cell concentrate (RCC) 1070 mL, fresh frozen plasma (FFP) 720 mL, and platelet concentrates (PC) 400 mL was required. Intraoperative photo is presented (Figure 2).

Postoperative rebleeding was not observed (Figure 1C). The Impella was weaned and explanted 42 days after the AMI onset. The patient was transferred to a rehabilitation hospital, and severe dysarthria, mild motor aphasia, and right hemiparesis persisted for three months after hematoma evacuation, and the symptoms remained during follow-up.

Case two

A 54-year-old male was transferred to our hospital with an ECG from a previous hospital showing anterior wall AMI. The patient lost consciousness and developed ventricular fibrillation. Cardiopulmonary resuscitation, intubation, mechanical ventilation, and repeated electrical defibrillation were performed immediately. The patient was considered to have cardiogenic shock and cardiac arrest due to AMI, which are the indications for venoarterial extracorporeal membrane oxygenation (VA-ECMO) and Impella. Emergency PCI for complete occlusion of the left anterior descending artery was performed using the Impella and VA-ECMO placements (Figure 2A).

**Figure 3:**
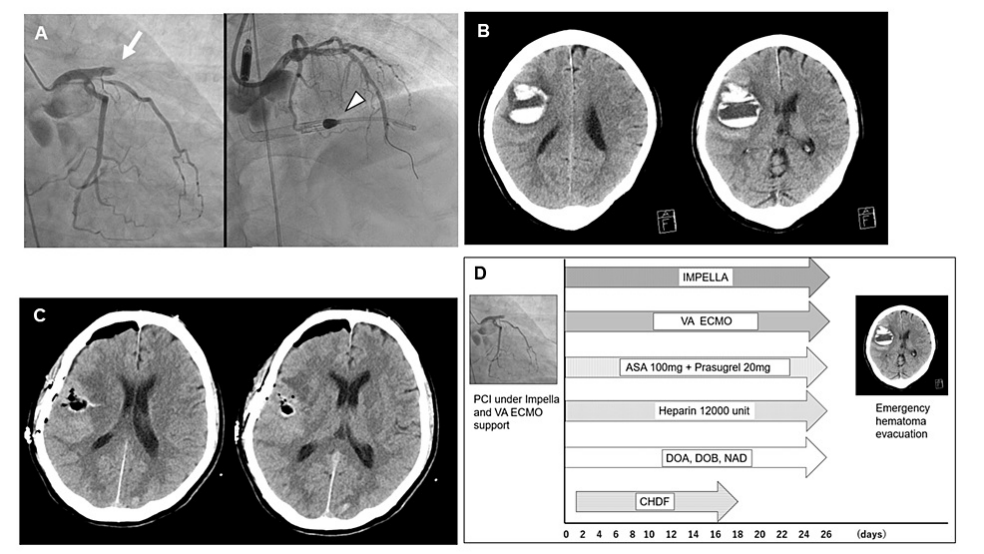
Clinical course of case two A) Images of left anterior descending artery before (left) and after (right) percutaneous coronary intervention:  Complete occlusion of left anterior descending artery was treated by percutaneous transluminal angioplasty and stent placement. Impella was implanted to treat cardiogenic shock; B) Intracerebral hemorrhage in the left frontal and temporal lobes: The hematoma volume is 59.3 mL; C) Computed tomography after emergency hematoma evacuation shows the removal of hematoma; D) Graph showing the treatment process of case two from admission to onset of intracerebral hemorrhage. VA ECMO - venoarterial extracorporeal membrane oxygenation; PCI - percutaneous coronary intervention; ASA - acetylsalicylic acid

PCI resulted in thrombolysis in myocardial infarction (TIMI) II. Dual antiplatelet therapy (ASA, 100 mg; prasugrel, 20 mg) and heparinization were administered, followed by PCI. ECG showed poor ventricular wall motions. Refractory hemolysis, acute renal failure, and anemia were observed two days after PCI, and haptoglobin injection and continuous hemodiafiltration (CHDF) was initiated (creatinine 2.19). The patient's condition and consciousness gradually improved, and VA-ECMO was weaned seven days after PCI. The patient experienced consciousness disturbance, conjugate deviation, and left hemiparesis 26 days after the PCI. CT revealed right intracerebral hemorrhage (ICH; hematoma volume: 43.2 mL; Figure 2B). An emergency surgical hematoma evacuation was performed on the same day by a certified neurosurgeon. Intraoperative blood loss was 380 mL; however, transfusion was not necessary. Postoperative rebleeding was not observed (Figure 2C). Figure 2D shows the treatment process from admission to ICH onset. CHDF and Impella were weaned at 33 and 112 days, respectively, after PCI. The patient was transferred to a rehabilitation hospital with severe dysarthria and left hemiparesis 4 months after the hematoma evacuation. Figure 1D shows the treatment process from admission to ICH onset.

Case three

A 52-year-old male with dyspnea on exertion and hypotension was transferred to our hospital because of poor hemodynamic status despite intensive medical treatment in another hospital. Cardiac ultrasonography revealed decreased systolic function (Figure 3A).

**Figure 4:**
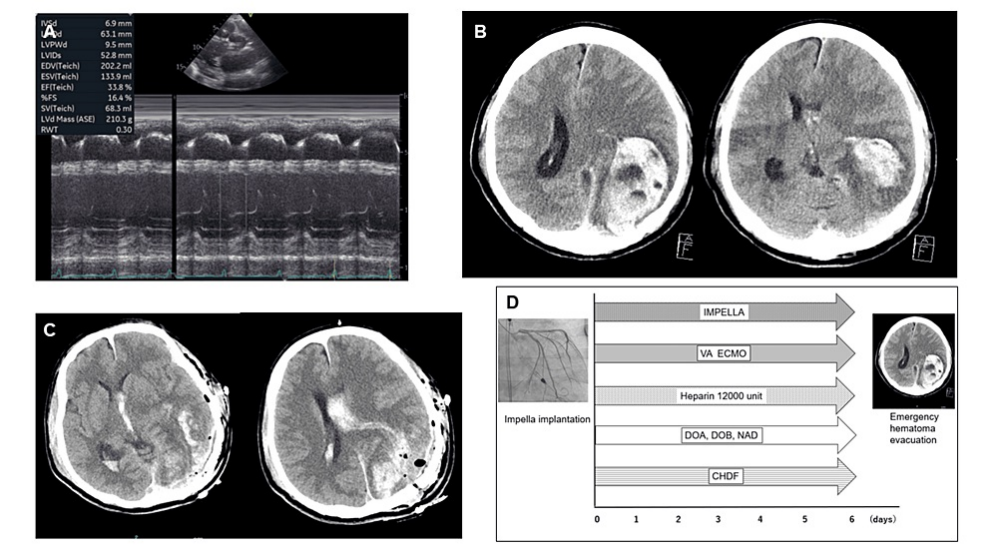
Clinical course of case 3 A) Cardiac ultrasonography showing decreased systolic function; B) Intracerebral hemorrhage in the left parietal and occipital lobes. The hematoma volume is 62.8 mL; C) Computed tomography showing the removal of hematoma; D) Graph showing the treatment process of case three from admission to the onset of intracerebral hemorrhage. VA ECMO - venoarterial extracorporeal membrane oxygenation; PCI - percutaneous coronary intervention; ASA - acetylsalicylic acid

Despite high-dose inotropes and vasopressors, hypotension and cardiovascular failure persisted (lactate, 12 mmol/L). Cardiac catheterization revealed an average pulmonary artery pressure of 29 mmHg, pulmonary artery wedge pressure of 25 mmHg, left ventricular end-diastolic pressure of 25 mmHg, and cardiac output of 1.83 L/min. No coronary artery stenosis was observed. Based on these clinical data, the patient was diagnosed with dilated cardiomyopathy. Metabolic acidosis (pH 7.255) and acute renal failure (creatinine 3.78) were detected, and multiple organ failure was diagnosed using blood gas and venous blood analyses. Impella CP and VA-ECMO were implanted to maintain hemodynamic stability and reduce the cardiac load for three days, followed by which the patient developed anisocoria and became comatose. CT revealed parietal and occipital subcortical hematomas (hematoma volume: 62.8 mL; Figure 4B). Protamine has been used as the principal neutralizing agent of heparin. Emergency hematoma evacuation was performed. Intraoperative findings revealed oozing from the epidural and subdural spaces, which made the bleeding difficult to control. Intraoperative blood loss was 3205 mL, and transfusion was required (RCC, 1020 mL; FFP, 960 mL; and PC, 200 mL). Postoperative CT revealed oozing and residual hematoma (Figure 4C). Figure 4D summarizes the treatment process from admission to ICH onset. After surgical evacuation, the cardiac shock and multiple organ failure did not improve. The patient died 14 days after admission.

## Discussion

We presented three cases of ICH that occurred during Impella ventricular assist device placement for the management of cardiogenic shock. Surgical hematoma evacuations were performed; however, the intraoperative blood loss was higher than usual, necessitating platelet and FFP transfusions. Platelet infusion is effective for patients with antiplatelet agents. FFP transfusion is useful to replenish the lost coagulation factors. To our knowledge, this is the first report of patients with ICH requiring surgical hematoma evacuation while using an Impella support device.

Currently, Impella is widely used to treat various heart failure conditions. Patients with advanced heart failure are increasingly placed in acute mechanical circulatory support (MCS) systems while awaiting transplantation [7]. Similarly, in the treatment of ischemic heart disease, temporary support is used to a greater degree for high-risk PCIs [6,8]. Although Impella drastically improves the survival of patients with severe heart failure, thrombotic and bleeding complications can cause serious morbidity and mortality [1,5,6,9-11]. Miyashita et al. reported stroke occurrence in 2.1% of patients with Impella placement in their meta-analysis [9]. In our experience, Impella was used for 62 patients with cardiac shock during presented three ICHs. Hassett et al. reported their clinical experience of three ischemic strokes, two intracerebral hemorrhages, and one subdural hematoma in 79 patients with short-term Impella placement [5]. They suggested the attribution of hemorrhagic events to anticoagulant use and thrombocytopenia and claimed that ICHs are not uncommon during short-term cardiac support periods with Impella. The average duration from Impella to ICH occurrence was comparative short-term was 6.0 days in a previous report [5]. 

The lack of sufficient studies has made it difficult to establish clear criteria for surgical hematoma evacuation during Impella-assisted device placement. As coagulopathy is known to occur under the Impella assist device, urgent surgical management and reversing of heparin are recommended to reduce mortality and morbidity [4]. Surgical evacuation can be lifesaving in selected patients with hemorrhage and rapid progression of neurological deficits. Anesthesiologists should have plenty of knowledge about Impella and preparation for shock vital due to intraoperative blood loss.

In our three cases, the location of hematoma was subcortical. It is still obscure if coagulopathy relates to the location of hematoma. Further study is mandatory.

In our patients, the intraoperative blood loss was much higher than that in the usual hematoma evacuation. The possible causes include the administration of dual antiplatelet therapy post PCI (cases one and two), heparinization to prevent biomaterial build-up and subsequent device dysfunction (cases one, two, and three) [1,4], and acquired von Willebrand syndrome, a known complication of the Impella device that causes bleeding complications [11,12]. The mechanism of acquired von Willebrand syndrome in the patient with Impella was thought of as shear stress-induced conformational change and subsequent proteolytic cleavage [12]. Platelet aggregation test or vWF:RCo assay was not performed in our patients because hematoma evacuation was performed as an emergency surgery. It is usually difficult to determine whether a bleeding tendency exists during emergency surgery. Therefore, in cases of emergency surgical hematoma evacuation when using the Impella device, neurosurgeons must keep in mind that platelet and FFP transfusion and reversal of heparin may be required for heavy intraoperative bleeding [4]. In case three, surgical hematoma evacuation was performed in the supine position with head rotation, even when the hematoma was present in the parieto-occipital lobe. It is difficult to perform hematoma evacuation in the prone position because the tip of the Impella is easy to move, resulting in no cardiac flow. Surgery should be performed in the supine position, with the head and bed rotated.

Along with the increasing number of patients undergoing MCS implantation to treat cardiogenic shock, the number of patients requiring hematoma evacuation, as in the present cases, might increase.

## Conclusions

ICH is a potential cause of morbidity associated with Impella placement because of muti-anticoagulants, antiplatelet agents, and coagulopathy. Surgical hematoma evacuation is a lifesaving treatment. The amount of intraoperative blood loss tended to be higher than that in usual hematoma evacuation. In cases of emergency surgical hematoma evacuation, when using the Impella device, platelets and FFP should be kept ready for transfusion in heavy intraoperative bleeding cases. Hematoma evacuation can be performed in the patients under the use of Impella if there is sufficient preparation of blood infusion.
